# Isolated lumbar extension exercise alone or in a multimodal program for low back pain and radiculopathy: a non-randomized controlled trial

**DOI:** 10.1038/s41598-025-22452-x

**Published:** 2025-10-23

**Authors:** Bruno Domokos, Julia Domokos, Gustav Andersson, Stefan Mannel, Linda May Weigel, Horst Josef Koch, Birgit Wallmann-Sperlich, Christoph Raschka, Christoph Spang

**Affiliations:** 1 Institute for Sports Science, University of Würzburg, Würzburg, Germany; 2 Private Orthopaedic Spine Center Dr. Alfen & Colleagues, Würzburg, Germany; 3 Department of Medical and Translational Biology, Umeå University, Umeå, Sweden; 4 Department of Diagnostics and Intervention, Hand and Plastic Surgery, Umeå University, Umeå, Sweden; 5 Innovative Manual Therapy Mannel, Würzburg, Germany; 6 Heinrich-Braun-Klinikum, Zwickau, Germany; 7 Integrative and Experimental Exercise Science and Training, Faculty of Human Science, Institute for Sports Science, University of Würzburg, Würzburg, Germany

**Keywords:** Low back pain, Radiculopathy, Exercise therapy, Muscle strength, Diagnostic imaging, Ultrasonography, Phase II trials, Rehabilitation

## Abstract

Isolated lumbar extension resistance exercise (ILEX) has been shown to effectively address chronic low back pain (LBP) and paraspinal deconditioning. However, its role within the widely recommended multimodal management approaches remains unclear. This study aimed to: (1) closely monitor the effects of ILEX throughout the course of a 16-weeks intervention, applied as a stand-alone approach in patients with nociceptive and/or neuropathic pain and (2) to compare this intervention with a multimodal treatment program including general exercise (GE) and manual therapy (MT). Fifty-eight LBP patients were enrolled in this single-center (Wuerzburg, Germany), prospective, non-randomized controlled trial. The ILEX-only group (*Powerspine Back* *[PSB]*: n = 29) completed 25 sessions of ILEX, whereas the integrative group (*PSB*+ : n = 29) also received GE (added to each session) and MT (5 to 7 sessions). Outcome measures were assessed at baseline, 3, 6, 9 and 16 weeks including lumbar multifidus cross-sectional area (CSA), muscle thickness (MT), echointensity (EI) (ultrasound-derived), isometric lumbar strength (isokinetic device) and validated questionnaires (Visual Analog Scale [VAS]; Oswestry Disability Index [ODI]; Short-Form 36). Between-subjects repeated measures ANOVA and correlation analyses were performed. Both groups (PSB: m = 16, f = 15, Ø40.26 (± 13.71) years, BMI 25.16 (± 4.07) kg/m^2^; PSB+ : m = 16, f = 13, Ø42.00 (± 12.69) years, BMI 25.20 (± 3.29) kg/m^2^) demonstrated comparable linear improvements in multifidus CSA (PSB: Δ0.59 [95% CI 0.36–0.82] cm^2^; PSB+ : Δ0.72 [95% CI 0.49–0.96] cm^2^, both *p* < 0.001; main effect (group): *p* = 0.52) along with similar strength gains (PSB: Δ96.79 [95% CI 57.64–135.93] Nm; PSB+ : Δ86.88 [95% CI 48.70–125.06] Nm; both *p* < 0.001; main effect (group): *p* = 0.91) over time. EI remained unchanged in both groups. No-between group differences were observed for self-reported measures, while all outcomes significantly improved (ODI: PSB: − 15.0 [95% CI − 19.3 to − 10.8], PSB+ : − 11.7 [95% CI − 16.0 to − 7.4]; VAS: PSB: − 29.30 [95% CI − 37.73 to − 20.86], PSB+ : − 29.34 [95% CI − 37.78 to − 20.91], both *p* < 0.001). Only PSB+ showed correlations between multifidus changes and clinical outcomes. Stand-alone and combined isolated lumbar extension resistance exercise interventions are effective in reversing muscle deconditioning and produce comparable clinical outcomes. The results inform clinical decision-making and support the development of targeted, resource-efficient rehabilitation strategies.

*ClinicalTrials.gov Identifier* NCT06890052 (20/03/2025) (https://clinicaltrials.gov/study/NCT06890052?cond=NCT06890052%20&rank=1)

**Supplementary Information:**

The online version contains supplementary material available at 10.1038/s41598-025-22452-x.

## Introduction

Low back pain (LBP) is the leading cause of disability worldwide significantly impacting well-being, quality of life and daily functioning of those affected^1–3^. While it is acknowledged that the origin of chronic low back pain (CLBP) is multifactorial, the exact pathological mechanisms are still not completely understood^4^. Recent guidelines, such as those by the World Health Organization outline a range of treatment options—including exercise therapy, education, manual therapy (MT), psychological interventions, and, in some cases, pharmacological support^5^. While the individual contribution of each approach remains uncertain, multimodal and patient-centred strategies appear to be the most promising overall^5^. The lack of specificity in current guidelines and the broad application of multimodal interventions underscore the need for further research into both isolated and combined approaches to gain deeper insight into the mechanisms of pain onset, persistence, and rehabilitation^2^.

Research on chronic low back pain (LBP) increasingly focuses on the multifidus (MF) muscle, a key stabilizer of the spine^6^. Factors such as inactivity, degeneration, and microtrauma contribute not only to pain onset but also to a loss of neuromuscular control and functional impairment, collectively known as “multifidus dysfunction”^7,8^. This dysfunction often results from injury-induced arthrogenic muscle inhibition, nerve compression, inflammation, and motor control deficits, leading to muscle deconditioning, including atrophy and functional decline^7–9^. Interestingly, imaging studies have shown that MF alterations—such as reduced muscle size, fat infiltration, and impaired function—occur not only in nonspecific LBP but also in specific lumbar conditions like facet joint arthritis^10^, spondylolisthesis^11^, spinal stenosis^12^, and disc degeneration with radiculopathy^13^. In chronic stages, when atrophy and dysfunction are advanced, targeted exercise therapy becomes especially important^14^.

Isolated lumbar extension exercise (ILEX) is designed to target the paraspinal extensor muscles and has shown to be clinically effective in several studies^15,16^. It is widely implemented in many countries, predominantly in Germany, following the guidelines of the German Medical Strengthening Association (GMKT)^17^. An empirically well-supported rationale of ILEX is that strength gains are correlated with clinical improvements^16^. However, several gaps in the current literature obscure its potential and limit its application in clinical settings. Firstly, reviews and meta-analyses conducted on ILEX reveal that almost all prior studies have not simultaneously assessed structural, functional and clinical outcomes^15,16^. As a result, critical questions remain unanswered regarding the specific physiological and functional changes that occur during interventions aimed at reversing muscle deconditioning. Secondly, most studies have been conducted on nonspecific LBP populations^15,16^. This contrasts with the growing demand for individualized exercise interventions, which are more appropriately developed using more homogeneous patient groups like those with identifiable nociceptive or neuropathic origins^18,19^. In a retrospective analysis, Golonka et al. showed that patients with specific disc pathologies achieved good clinical outcomes with ILEX, while also emphasizing the need for prospective studies in well-defined homogenous patient cohorts^19^. Finally, Fortin et al. were the first to simultaneously investigate functional and morphological adaptations resulting from ILEX in LBP^20^. Their study evaluated a combined intervention of ILEX and motor control training versus general exercise (GE), reporting improvements in muscle size, function and clinical outcomes only in the ILEX group. However, the authors’ suggested future directions highlight the necessity to determine which specific type of exercise is most effective- such as ILEX as a stand-alone intervention^20–22^. Beyond advancing the understanding of the underlying rehabilitation mechanism of ILEX, it is equally important from a clinical perspective to clarify how ILEX can be most effectively integrated into multimodal management recommendations.

Against this background, this study aims to address existing gaps in the evidence base for ILEX by pursuing two primary objectives**:** (1) to closely monitor the effects of ILEX throughout the course of an intervention, applied as a stand-alone approach in a patient cohort with clear indicators of nociceptive and/or neuropathic pain; and (2) to compare this standalone ILEX intervention with a multimodal treatment program that includes additional general exercise (GE) and manual therapy (MT). The findings are intended to inform scientific understanding and clinical decision-making, and to support the development of more targeted, resource-efficient rehabilitation strategies that are feasible for clinicians and meaningful for patients.

## Materials and methods

### Design and setting

This trial was a two-arm, prospective, non-randomized study (NCT06890052; 20/03/2025) with a 1:1 allocation ratio and a repeated-measures design, investigating the clinical effectiveness of ILEX and the associated structural and functional adaptations, compared with an integrative multimodal treatment approach including ILEX. Outcomes were assessed at five points over the 16-week intervention period (0-, 3-, 6-, 9-, 16-weeks). The study was conducted in a single facility, at a private orthopedic spine clinic in Wuerzburg (Germany) in cooperation with Julius-Maximilians-University (Wuerzburg, Germany). Ethics approval was received by the same institution (ID: 1/2023). All participants signed informed consent.

### Participants, patient allocation and sample size

Patients were recruited from the regular practice operations between 12/2021 and 01/2024 and asked if they would voluntarily participate in the study. The following inclusion criteria had to be met for eligibility: (a) symptoms between the gluteal fold and the lower thoracic spine including leg pain associated with nerve root compression due to lumbar disc herniation (disc prolapse, ICD-10: M51.1, M51.2) or other spinal pathologies (e.g., spondylolisthesis, spondylarthritis) (ICD-10: M43.1, M47.2, M54.1, M54.4), (b) mild to moderate pain intensity (visual analog scale (VAS) range 25–75 at the time of the clinical assessment), (c) pain duration over 3 months with no other primary pain (e.g., neck pain), (d) between 18 and 65 years of age. Spine-specific red-flag conditions (e.g., spinal tumor, cauda-equina syndrome) and other conditions contraindicating participation in an exercise intervention (acute back pain, pregnancy, severe cardiovascular disease) were considered exclusion criteria (Table A1, appendix). The use of medication (e.g., pain killers) was recorded in a separate intake form. For ethical and organizational reasons, group allocation was not randomized but based on patient preference. All patients attended the facility specifically for ILEX-based therapy. While the multimodal approach (including general exercise and manual therapy) represents standard care at the facility, patients were given the option to undergo ILEX as a stand-alone intervention for research purposes. This allowed participants to choose between a stand-alone or a combined approach, while alternative randomization-based strategies were not feasible in this clinical setting. Regarding blinding, outcome assessments were performed by two assessors, while the exercise interventions (ILEX and general exercise) were delivered by multiple therapists who could not be blinded to group allocation due to the nature of exercise interventions^20^. The same two assessors, together with one supervisor, conducted the outcome analyses, all blinded to group allocation. No formal a priori sample size calculation was performed as no external reference values for the primary outcome were available at the time of study planning. Accordingly, participant recruitment followed a pragmatic feasibility-driven approach, focusing on the identification of thoroughly assessed patients meeting specific ICD criteria, presenting during the study period. For transparency, a post-hoc sample size/power calculation for the primary outcome was performed using Gpower 3.1^23^ and is reported in the results. Additionally, healthy volunteers without LBP during the previous 12 months were recruited from local companies via e-mail and through advertisements on the facility’s social media channel for the purpose of collecting reference data.

### Procedures

#### Clinical evaluation

Before the therapy, the patient underwent a 45-min, systematically structured, clinical evaluation^24^ conducted by one of the three physicians working in the facility: (1) an orthopedic physician and neurosurgeon (30 years of experience), (2) a neurosurgeon (30 years of experience) and (3) an orthopedic physician (6 years of experience) to assess the patient’s medical history, clinical signs, and relevant imaging findings. If magnet resonance imaging (MRI) or computed tomography (CT) was available, findings were discussed to evaluate the condition of passive structures (e.g., intervertebral discs, facet joints) and active structures (e.g., muscle morphology) in the lumbar region. Additionally, various mobility and neurological tests (e.g., Lasègue test, Bragard test, etc.) were conducted to assess potential nerve compression issues (e.g. sciatica) along with muscle strength tests of the lower extremity muscles and sensory examinations for hypoesthesia and paresthesia^24^. Based on the assessment and any prior diagnostic findings, specific conditions (e.g., disc herniation, spondylarthritis, spondylolisthesis) were documented in the patient’s medical record. Additional medical evaluations were scheduled after three, six, twelve, eighteen and twenty-five sessions. No serious harms were expected. In cases of sustained worsening of symptoms or disease progression, patients received additional consultations, and the protocol was adapted or alternative treatment options (e.g., surgical evaluation) were pursued.

#### Exercise intervention

The therapy program followed the guidelines of the GMKT^17^ and lasted 16 weeks, with the first 18 sessions being performed twice per week, followed by seven sessions once per week. Two experienced evaluators with degrees in sports science, who had also been involved in previous reliability studies^25^, conducted the assessments.

Aside from assessment days, all exercise sessions, including the specific procedure on the *Powerspine Back* (PSB) ILEX device (nr. 30000-367, Powerspine, Wuerzburg, Germany), were supervised by qualified personnel, primarily sports scientists at undergraduate or graduate level. Manual therapy sessions were carried out by personnel specified in accordance with the principles of the institute for applied manual therapy (IFAMT)^26^. All participants were asked to refrain from any additional therapeutic interventions (e.g., physiotherapy or massages).

### Intervention program 1 (PSB-only)

For the ILEX-intervention, the same procedure was followed as described in earlier studies^25^: Prior to the exercise, the device was manually adjusted by the therapist to correspond with the participant’s anthropometric characteristics. This included the adjustment of the pelvic restraint system consisting of a foot board, a knee pad, a thigh belt and a variable seat cushion. To ensure biomechanics maximizing the activation of the lumbar extensor muscles while minimizing engagement of the hip and lower limb muscles, the patient was positioned in a semi-sitting position with maximum tolerable fixation^27^. Afterward, the counterweight was calibrated to provide uniform resistance during the movement. The exercise protocol also followed the GMKT guidelines^17^ with the initial sessions focusing on submaximal training intensity and biofeedback provided by a visual panel on a computer screen via software. Each repetition of the flexion–extension cycle was standardized to exactly 10 s, consisting of two dynamic phases (four seconds for flexion and extension) and an isometric hold in extension (two seconds). The protocol can be divided into two phases, a submaximal adaptation phase, and a maximum exertion strengthening phase: Following a gradual progression in resistance, the program aimed for maximum exertion no earlier than after 8–12 sessions. By this point, a patient should reach voluntary muscular failure after approximately 12 repetitions (equalling a time under tension of 120 s). Additionally, the device’s software suggests an individualized range of motion tailored to the patient’s pain comfort and diagnoses defined in the clinical evaluation, typically ranging between 10° and 50° depending on the severity of the condition (Figure A1, appendix).

### Intervention program 2 (PSB + GE + MT)

The same ILEX-protocol was applied to the group receiving additional therapeutic components. The supplementary exercise program (GE) focused on strengthening the core stabilizing muscles of the abdomen, hips, and gluteal region, as well as larger back muscles such as the latissimus dorsi and rhomboids. Exercises were individually prescribed, with muscle group prioritization tailored to each patient’s symptoms and underlying pathology. For example, the focus for a patient with spondylolisthesis was on stabilizing the core with specific exercises like the abdominal crunch machine. In contrast, a patient with radicular symptoms should avoid movements that exacerbate pain (such as forward flexion in the abdominal crunch), instead emphasizing postural control in neutral positions such as during the latissimus pulldown. Everyone’s program was standardized to four additional exercises (Table A2, appendix), each performed for one set of approximately two minutes under tension, with a focus on optimal movement control.

The manual therapy program according to IFAMT^26^ is considered as an evidence-based approach for the treatment of musculoskeletal disorders, combining both passive and active components. Each therapist utilizes a comprehensive range of techniques, including the treatment of paraspinal soft tissues (e.g., joint mobilization, myofascial release, trigger point therapy), neurodynamic techniques (e.g., nerve gliding exercises and nerve mobilization), and spinal decompression (e.g., manual traction). In addition to functional goals such as restoring mobility and improving functional movement patterns, the program also aims for both short- and long-term pain relief by equipping the patient with self-help management tools including home-based exercises. During the first 18 sessions of the ILEX protocol, MT is scheduled five times (once per week). Three additional sessions are scheduled in the phase from PSB sessions 18 to 25. The first session was scheduled for 60 min including an oral assessment of patient history while the following sessions lasted for 30 min.

### Testing battery and outcome measures

Assessment sessions started with a brief consultation regarding the patient’s current condition and a standardized application of the measurements in the following order: (1) ultrasound (GE Healthcare, Logiq S7 Expert, nr. 5460683), (2) self-reported questionnaires (Visual analog scale, Oswestry disability index, Short-Form 36), (3) isometric strength tests (Powerspine Back isokinetic device). Each measurement was conducted according to protocols tested for reliability in previous studies^25^. For detailed ultrasound procedures including standardization strategies (patient orientation, digital probe angulation, etc.) and reliability indices see also Domokos et al.^25^. Analysis of ultrasound images was performed using ImageJ software and was conducted by one assessor. Maximum isometric strength test was only tested if the condition allowed. All outcome measures were obtained at baseline, after 3 weeks (before the 6th session), 6 weeks (before the 12th session), 9 weeks (before the 18th session) and 16 weeks (before the 24th session). Only instruments that are well established in the field and extensively tested for psychometric properties were used. Table 1 provides an overview of the primary and secondary outcomes including references for psychometric properties.

**Table 1:** Outcome measures (unit) and description of measurement properties, including references relating to reliability and validity.

Outcome measure		Measurement properties
Primary outcome		
Morphology (ultrasound-derived)^25,28^	Multifidus cross-sectional area (CSA, cm^2^)	Multifidus muscle cross-sectional area measurements are obtained from ultrasound (US) from the L4/L5 spinal level (left and right)
	Multifidus muscle thickness (MT, cm)	Multifidus muscle thickness is measured with ultrasound (US) from the L4/L5 spinal level (left and right)
	Multifidus echo intensity (EI, 0–255 AU)	Multifidus echo intensity (EI) is measured with ultrasound (US) from the cross-sectional area of the L4/L5 spinal level (left and right). EI is measured on a scale from 0–255 in arbitrary units (ImageJ)
Secondary outcomes		
Clinic (patient-reported)^29–31^	Pain intensity (mm, 0–100)	Pain intensity is measured with the visual analog scale (VAS) on a rating scale from 0 – 100
	Health-related quality of life (pts., 0–100)	Health-related quality of life (QoL) is measured with the short-form survey 36 (SF-36). The survey consists of 8 domains assessing physical and mental sum scores (SF-P and SF-M). Scores in each domain can range from 0 (zero health) to 100 (perfect health)
	Disability (pts., 0–50)	Disability concerning LBP is measured with the Oswestry Disability Index (ODI). It includes 10 items (e.g., sitting, standing, personal care) with a range from 0–5 with higher scores indicating greater disability
Function (isokinetic machine)^32^	Isometric lumbar extensor muscle strength (Nm)	Isometric lumbar extensor strength is measured with an isokinetic dynamometer included in the Powerspine Back ILEX device. Measurements were taken at 39°, 30°, 24° and 15°

### Statistical analysis

The statistical analysis was conducted as described in previous studies^20^. Descriptive statistics included means and standard deviations for all outcome measures in each group as well as compliance (number of total sessions attended) and adherence (sessions attended as scheduled) rates were collected. Data were tested for normal distribution using the Kolmogorov–Smirnov test. One-way ANOVAs were performed to assess differences between the three groups at baseline for parametric comparisons, and the Kruskal–Wallis-Test for non-parametrical comparisons. Changes in outcomes over time were assessed using repeated measures ANOVA, with baseline values included as covariates to adjust for potential differences between groups, and including group, time and their interaction effects.

Analyses were conducted per-protocol, including only participants who competed all scheduled measurements (in particular: primary outcome). No imputation of missing data was performed. Serious harms, defined as incidences preventing a participant from completing the intervention, were analyzed descriptively.

Pre-specified additional analyses included correlations between changes in primary and secondary outcomes. Pearson’s correlation was used with effect sizes categorized according to Cohen’s classification (small 0.10., moderate 0.30., strong 0.50). All analyses were conducted using IBM SPSS (v26), with a significance level set at *p* < 0.05.

## Results

### Sample demographics, baseline values and adherence

A total of 1.852 patients were accessed for eligibility and 72 participants meeting the inclusion criteria were enrolled after doctoral assessment (Figure A2, appendix), which provides ≥ 80% power under the assumptions of our post-hoc sample size calculation: The sample size calculation for the primary outcome (multifidus CSA) using Gpower 3.1 (repeated-measures ANOVA, within-between interaction, σ = 2, expected difference = 0.6 cm^2^^20,25^, ρ = 0.6^33^, number of measurements: r = 5, σ = 0.05, 1-β = 0.8, 10% anticipated dropout) yielded an effective required sample of n = 49^23^. Additionally, 32 healthy participants (m = 17; f = 15) volunteered to be screened with the diagnostic instruments to generate reference data.

A total of 37 participants decided to enroll in the PSB standalone group and 35 participants favored the integrative PSB+ program including general exercise and manual therapy (PSB+). Four patients (PSB: n = 2, PSB+ : n = 2) were initially enrolled but excluded from the final analysis because their pain levels decreased below the required threshold by the start of the intervention. Ten participants (PSB: n = 6, PSB+ : n = 4) dropped out during the study for personal reasons (e.g., private circumstances) or by assessor withdrawal to ensure high adherence. Regarding adverse events, one participant in the PSB+ group experienced aggravated pain, leading to study discontinuation and subsequent surgery.

In sum, 29 patients in each group (PSB: m = 15, f = 14; mean age 40.26 (± 13.71) years, BMI 25.16 (± 4.07) kg/m^2^; PSB+ : m = 16, f = 13; mean age 42.00 (± 12.69) years, BMI 25.20 (± 3.29) kg/m^2^) completed all five assessments to be eligible for inclusion in the final analysis. More than half of the participants in each group had a previous MRI (PSB: n = 18; PSB+ : n = 16). Compliance was comparable with participants in the PSB group having attended 23.26 (± 1.21) sessions (93.0%), while those in the PSB+ group attended 23.13 (± 0.81) sessions (92.5%). The intervention was completed on average after 17.0 (± 2.8) weeks in the PSB group and 18.7 (± 3.5) weeks in the PSB+ group, indicating high adherence to the presecribed protocol in both groups.

There were no significant differences in anthropometric data between the patient groups and the healthy control group. The SF Physical and SF Mental scores were significantly lower in the patient groups than in the healthy group (*p* < 0.001). Due to the absence of pain, VAS and ODI scores were only obtained for patient groups with no significant difference at baseline (*p* > 0.05). Mean strength values in healthy individuals were higher in all tested angles but only significantly higher in flexion (15°) (*p* < 0.05). Echo intensity (EI) was significantly higher in the healthy control group (*p* < 0.05) (Table A3, appendix).

### Effect and development over time of morphological outcomes in PSB standalone approach (PSB) and integrative approach (PSB+)

**Fig. 1:**
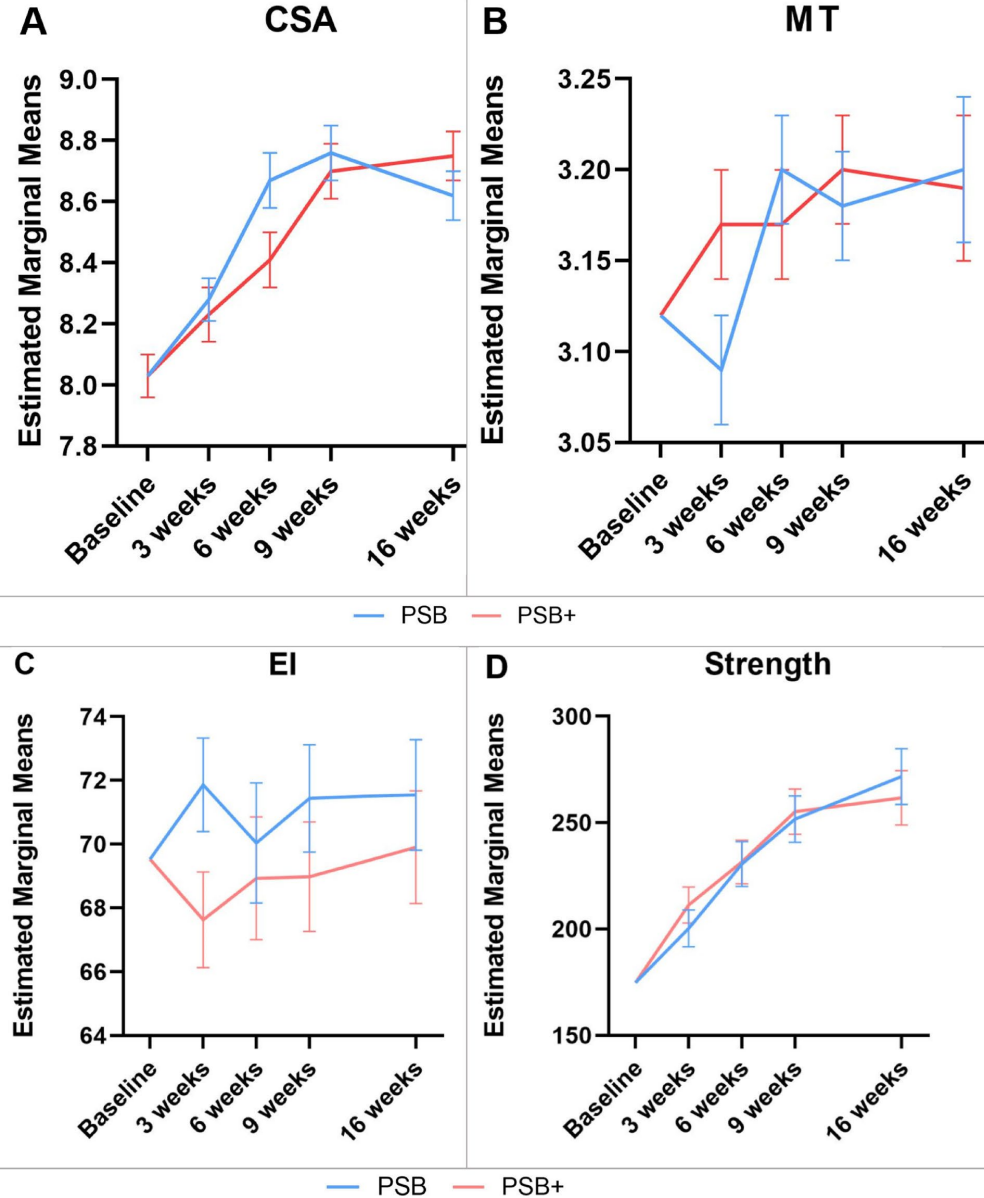

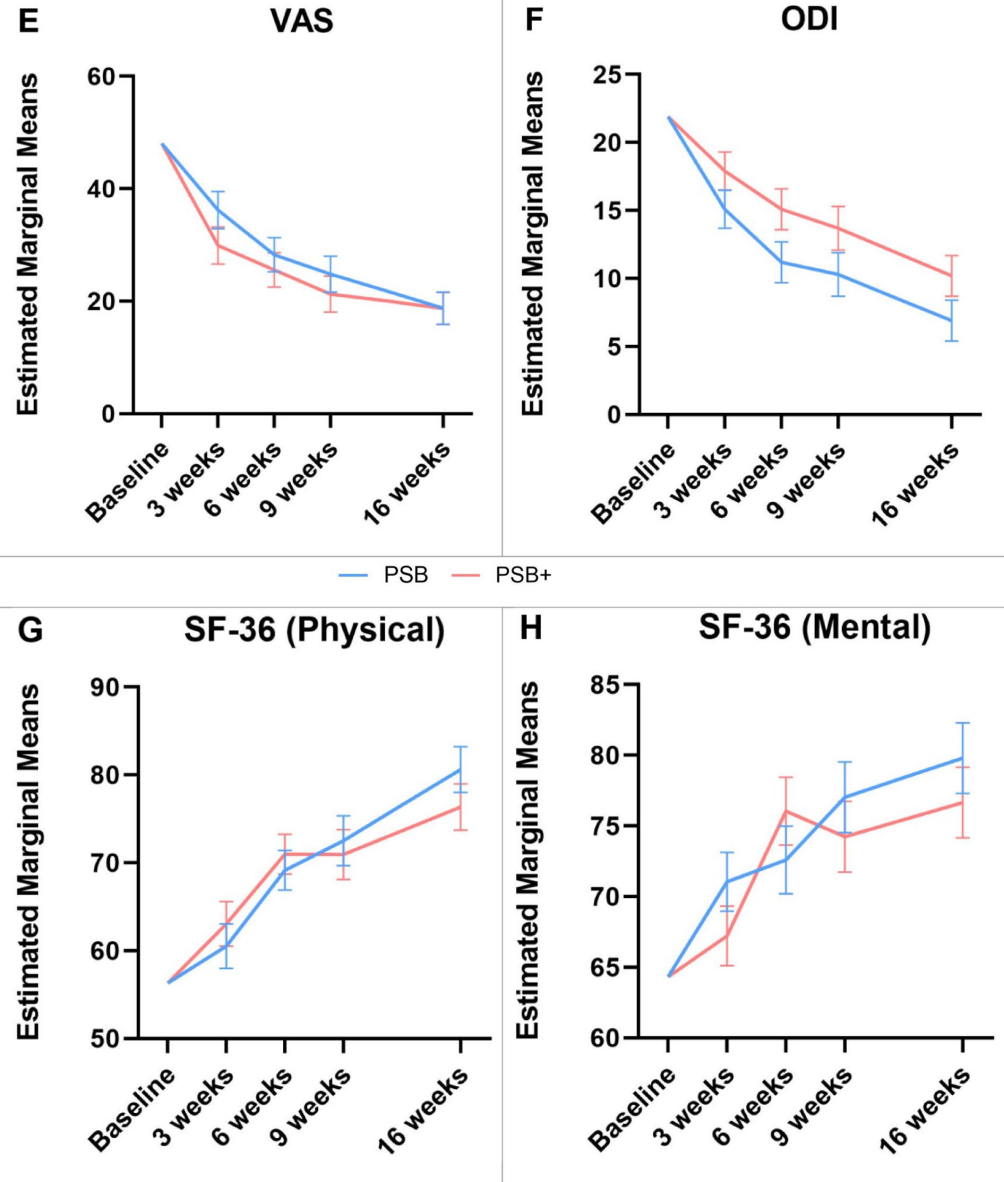
Line graphs of the mean changes over the intervention period in PSB and PSB+ . Changes in multifidus cross-sectional area (CSA, **A**), muscle thickness (MT, **B**) echo intensity (EI, **C**), strength (**D**), pain levels (VAS, **E**), disablility (ODI, **F**) and quality of life (SF-36 Physical, **G**; SF-36 Mental, **H**) in both groups, including standard error, adjusted for baseline values. Y-values respresent CSA in cm^2^, MT in cm, EI in arbitrary units, 0–255 AU, strength in Nm, pain intensity in mm (0–100), disability in points (0–50) and quality of life in points (0–100).

For multifidus CSA (total), the repeated measures ANOVA revealed a significant time*group interaction (*p* < 0.05, η^2^p = 0.17 ) (Table 2). In contrast, between-group differences were not significant (*p* = 0.52. η^2^p = 0.008). Postintervention, both groups showed a marked increase in CSA over the course of the therapy (PSB: mean difference [95% CI] 0.59 [0.36–0.82] cm^2^, *p* < 0.001; PSB+ : mean difference [95% CI] 0.72 [0.49 to 0.96] cm^2^; *p* < 0.001) (Fig. 1; histograms of absolute changes in outcome measures: Fig. A3, appendix).

**Table 2:** Adjusted morphological outcomes for the PSB and the PSB+ group.

Variables	Measurement period	PSB n = 29	PSB+ n = 29	Main effect of group	Interaction effect between time and group
CSA (r) (cm^2^)	Baseline	8.09	8.09	*p*-value = 0.61 F = 0.26 df = 1 η^2^p = 0.005	*p*-value = < 0.05 F = 4.48 df = 4 η^2^p = 0.26
	3 weeks (std. err)	8.24 (0.10)	8.36 (0.10)		
	6 weeks	8.59 (0.11)	8.37 (0.11)		
	9 weeks	8.62 (0.10)	8.69 (0.11)		
	16 weeks	8.86 (0.11)	8.65 (0.11)		
	MD (95% CI)	0.77 (0.46 to 1.08)	0.56 (0.25 to 0.88)		
	Main effect of time	*p*-value < 0.001	*p*-value < 0.001		
		F = 15.30	F = 9.95		
		df = 4	df = 4		
		η^2^p = 0.545	η^2^p = 0.438		
CSA (l) (cm^2^)	Baseline	7.97	7.97	*p*-value = 0.69 F = 0.16 df = 1 η^2^p = 0.003	*p*-value = < 0.001 F = 6.06 df = 4 η^2^p = 0.32
	3 weeks (std. err)	8.30 (0.08)	8.11 (0.09)		
	6 weeks	8.74 (0.11)	8.46 (0.11)		
	9 weeks	8.90 (0.11)	8.72 (0.11)		
	16 weeks	8.38 (0.10)	8.84 (0.10)		
	MD (95% CI)	0.41 (0.13 to 0.69)	0.88 (0.60 to 1.16)		
	Main effect of time	*p*-value < 0.001	*p*-value < 0.001		
		F = 19.07	F = 23.23		
		df = 4	df = 4		
		η^2^p = 0.60	η^2^p = 0.65		
CSA (total) (cm^2^)	Baseline	8.03	8.03	*p*-value = 0.52 F = 0.42 df = 1 η^2^p = 0.008	*p*-value = < 0.05 F = 2.64 df = 4 η^2^p = 0.17
	3 weeks (std. err)	8.28 (0.07)	8.23 (0.07)		
	6 weeks	8.67 (0.09)	8.41 (0.09)		
	9 weeks	8.76 (0.09)	8.70 (0.09)		
	16 weeks	8.62 (0.08)	8.75 (0.08)		
	MD (95% CI)	0.59 (0.36 to 0.82)	0.72 (0.49 to 0.96)		
	Main effect of time	*p*-value < 0.001	*p*-value < 0.001		
		F = 20.60	F = 24.11		
		df = 4	df = 4		
		η^2^p = 0.62	η^2^p = 0.65		
MT (r) (cm)	Baseline	3.12	3.12	*p*-value = 0.84 F = 0.04 df = 1 η^2^p = 0.001	*p*-value = < 0.05 F = 2.83 df = 4 η^2^p = 0.18
	3 weeks (std. err)	3.08 (0.03)	3.16 (0.03)		
	6 weeks	3.19 (0.03)	3.16 (0.03)		
	9 weeks	3.16 (0.03)	3.18 (0.03)		
	16 weeks	3.21 (0.03)	3.16 (0.03)		
	MD (95% CI)	0.09 (0.00 to 0.19)	0.05 (− 0.05 to 0.14)		
	Main effect of time	*p*-value < 0.001	*p*-value = 0.21		
		F = 7.37	F = 1.53		
		df = 4	df = 4		
		η^2^p = 0.36	η^2^p = 0.11		
MT (l) (cm)	Baseline	3.13	3.13	*p*-value = 0.63 F = 0.23 df = 1 η^2^p = 0.004	*p*-value = 0.16 F = 1.72 df = 4 η^2^p = 0.18
	3 weeks (std. err)	3.10 (0.04)	3.19 (0.04)		
	6 weeks	3.21 (0.03)	3.18 (0.03)		
	9 weeks	3.20 (0.04	3.21 (0.04)		
	16 weeks	3.19 (0 (0.06)	3.21 (0.06)		
	MD (95% CI)	0.07 (− 0.11 to 0.24)	0.09 (− 0.09 to 0.26)		
	Main effect of time	*p*-value < 0.001	*p*-value = 0.301		
		F = 5.45	F = 1.25		
		df = 4	df = 4		
		η^2^p = 0.30	η^2^p = 0.09		
MT (total) (cm)	Baseline	3.12	3.12	*p*-value = 0.71 F = 0.14 df = 1 η^2^p = 0.002	*p*-value = 0.06 F = 2.38 df = 4 η^2^p = 0.16
	3 weeks (std. err)	3.09 (0.03)	3.17 (0.03)		
	6 weeks	3.20 (0.03)	3.17 (0.03)		
	9 weeks	3.18 (0.03)	3.20 (0.03)		
	16 weeks	3.20 (0.04)	3.19 (0.04)		
	MD (95% CI)	0.08 (− 0.03 to 0.19)	0.07 (− 0.04 to 0.18)		
	Main effect of time	*p*-value < 0.001	*p*-value = 0.15		
		F = 7.45	F = 1.75		
		df = 4	df = 4		
		η^2^p = 0.36	η^2^p = 0.20		
EI (r) (AU)	Baseline	68.46	48.46	*p*-value = 0.32 F = 1.01 df = 1 η^2^p = 0.02	*p*-value = 0.61 F = 0.67 df = 4 η^2^p = 0.05
	3 weeks (std. err)	70.31 (1.58)	66.81 (1.61)		
	6 weeks	68.36 (1.79)	67.29 (1.82)		
	9 weeks	69.80 (1.54)	68.28 (1.57)		
	16 weeks	70.53 (1.93)	68.68 (1.97)		
	MD (95% CI)	2.06 (− 3.59 to 7.72)	0.22 (− 5.54 to 5.97)		
	Main effect of time	*p*-value = 0.49	*p*-value = 0.80		
		F = 0.88	F = 0.41		
		df = 4	df = 4		
		η^2^p = 0.06	η^2^p = 0.03		
EI (l) (AU)	Baseline	70.59	70.59	*p*-value = 0.18 F = 1.84 df = 1 η^2^p = 0.03	*p*-value = 0.15 F = 1.76 df = 4 η^2^p = 0.12
	3 weeks (std. err)	73.49 (1.61)	65.08 (1.64)		
	6 weeks	91.90 (2.17)	65.96 (2.21)		
	9 weeks	73.29 (2.02)	65.34 (2.05)		
	16 weeks	72.67 (1.79)	67.34 (1.82)		
	MD (95% CI)	2.08 (− 3.16 to 7.32)	0.41 (− 4.93 to 5.75)		
	Main effect of time	*p*-value = 0.38	*p*-value = 0.43		
		F = 1.08	F = 0.97		
		df = 4	df = 4		
		η^2^p = 0.08	η^2^p = 0.07		
EI (total) (AU)	Baseline	69.53	69.53	*p*-value = 0.24 F = 1.39 df = 1 η^2^p = 0.03	*p*-value = 0.27 F = 1.34 df = 4 η^2^p = 0.10
	3 weeks (std. err)	71.86 (1.47)	67.63 (1.50)		
	6 weeks	70.04 (1.89)	68.93 (1.93)		
	9 weeks	71.44 (1.69)	68.98 (1.72)		
	16 weeks	71.55 (1.74)	69.90 (1.77)		
	MD (95% CI)	2.02 (− 3.06 to 7.10)	0.36 (− 4.81 to 5.54)		
	Main effect of time	*p*-value = 0.40	*p*-value = 0.61		
		F = 1.04	F = 0.69		
		df = 4	df = 4		
		η^2^p = 0.08	η^2^p = 0.05

For MT (total), the analysis showed a non-significant time*group interaction (*p* = 0.06, η^2^p = 0.16) and non-significant between-group differences (*p* = 0.71, η^2^p = 0.002). Postintervention, changes in MT were significant in the PSB-group (mean difference [95% CI] 0.08 [− 0.03–0.19] cm, *p* < 0.001) but not in the PSB+ group (mean difference [95% CI] 0.07 (− 0.04 to 0.18] cm, *p* = 0.15).

There were no significant changes or differences in any EI measure (time*group: *p* = 0.27, η^2^p = 0.10; between-group: *p* = 0.24, η^2^p = 0.03; PSB-group mean difference [95% CI] 2.02 [− 3.06–7.10], *p* = 0.40; PSB+ -group mean difference [95% CI] 0.36 [− 4.81–5.54], *p* = 0.61) while mean values in the PSB group were consistently higher (Table 2).

### Effect and development over time of patient-reported outcomes in PSB standalone approach (PSB) and integrative approach (PSB+)

For patient-outcomes VAS and ODI, there was no significant time*group interaction (VAS: *p* = 0.69, η^2^p = 0.04; ODI: *p* = 0.43, η^2^p = 0.07) or between-group differences (VAS: *p* = 0.38, η^2^p = 0.01; ODI: *p* = 0.07, η^2^p = 0.06) (Table 3). Postintervention, both groups demonstrated statistically significant reductions in VAS (PSB: mean difference [95% CI] − 29.30 [− 37.73 to − 20.86], *p* < 0.001; PSB+ : − 29.34 [− 37.78 to − 20.91], *p* < 0.001) and ODI values (PSB: mean difference [95% CI] − 15.0 [− 19.3 to − 10.8], *p* < 0.001; PSB+ : − 11.7 [− 16.0 to − 7.4], *p* < 0.001) in a linear-like manner (Fig. 1).The change in VAS exceeded the minimum clinically important difference (MCID) of 20 mm^34^. Similarly, the change in ODI was also at the upper end of the MCID range of 4–16 points^35^.

**Table 3:** Adjusted patient-reported outcomes for the PSB and the PSB+ group.

Variables	Measurement period	PSB-only n = 29	PSB + GT + IMT n = 29	Main effect of group	Interaction effect between time and group
VAS (pts.)	Baseline	48.09	48.09	*p*-value = 0.38 F = 0.78 df = 1 η^2^p = 0.01	*p*-value = 0.69 F = 0.56 df = 4 η^2^p = 0.04
	3 weeks (std. err)	36.24 (3.31)	29.94 (3.31)		
	6 weeks	28.27 (3.05)	25.57 (3.05)		
	9 weeks	24.80 (3.20)	21.27 (3.20)		
	16 weeks	18.79 (2.88)	18.74 (2.88)		
	MD (95% CI)	− 29.30 (− 37.73 to − 20.86)	− 29.34 (− 37.78 to − 20.91)		
	Main effect of time	*p*-value < 0.001	*p*-value < 0.001		
		F = 26.96	F = 27.70		
		df = 4	df = 4		
		η^2^p = 0.68	η^2^p = 0.68		
ODI (pts.)	Baseline	21.9	21.9	*p*-value = 0.07 F = 3.37 df = 1 η^2^p = 0.06	*p*-value = 0.43 F = 0.97 df = 4 η^2^p = 0.07
	3 weeks (std. err)	15.1 (1.4)	17.9 (1.4)		
	6 weeks	11.2 (1.5)	15.1 (1.5)		
	9 weeks	10.3 (1.6)	13.7 (1.6)		
	16 weeks	6.9 (1.5)	10.2 (1.5)		
	MD (95% CI)	− 15.0 (− 19.3 to − 10.8)	− 11.7 (− 16.0 to − 7.4)		
	Main effect of time	*p*-value < 0.001	*p*-value < 0.001		
		F = 29.32	F = 17.32		
		df = 4	df = 4		
		η^2^p = 0.69	η^2^p = 0.57		
SF-36 (Physical) (pts.)	Baseline	56.32	56.32	*p*-value = 0.90 F = 0.02 df = 1 η^2^p = 0.0003	*p*-value = 0.39 F = 1.05 df = 4 η^2^p = 0.08
	3 weeks (std. err)	60.50 (2.54)	63.06 (2.54)		
	6 weeks	69.14 (2.26)	70.98 (2.26)		
	9 weeks	72.50 (2.84)	70.94 (2.84)		
	16 weeks	80.63 (2.63)	76.34 (2.63)		
	MD (95% CI)	24.31 (16.63 to 31.96)	20.02 (12.39 to 27.70)		
	Main effect of time	*p*-value < 0.001	*p*-value < 0.001		
		F = 25.68	F = 19.16		
		df = 4	df = 4		
		η^2^p = 0.66	η^2^p = 0.60		
SF-36 (Mental) (pts.)	Baseline	64.33	64.33	*p*-value = 0.57 F = 0.32 df = 1 η^2^p = 0.006	*p*-value = < 0.05 F = 2.80 df = 4 η^2^p = 0.18
	3 weeks (std. err)	71.06 (2.1)	67.22 (2.1)		
	6 weeks	72.60 (2.4)	76.05 (2.4)		
	9 weeks	77.03 (2.5)	74.24 (2.5)		
	16 weeks	79.80 (2.5)	76.66 (2.5)		
	MD (95% CI)	15.47 (8.24 to 22.70)	12.33 (5.09 to 19.57)		
	Main effect of time	*p*-value < 0.001	*p*-value < 0.001		
		F = 11.26	F = 6.85		
		df = 4	df = 4		
		η^2^p = 0.46	η^2^p = 0.35		

Quality of life measures showed no time*group interaction for the physical sum score (SF-36 physical: *p* = 0.39, η^2^p = 0.08) but a significant interaction in the mental domain (SF-36 mental: *p* < 0.05, η^2^p = 0.18). There were no significant between-group effects (SF-36 physical: *p* = 0.90, η^2^p = 0.0003; SF-36 mental: *p* = 0.57, η^2^p = 0.006). A significant increase in both groups was observed in the physical SF-36 score (PSB: mean difference [95% CI] 24.31 [16.63–31.96], *p* < 0.001; PSB+ : mean difference [95% CI] 20.02 [12.39–27.70], *p* < 0.001) and the mental SF-36 score (PSB: mean difference [95% CI] 15.47 [8.24–22.70], *p* < 0.001; PSB+ : mean difference [95% CI] 12.33 [5.09–19.57], *p* < 0.001) (Table 3). The improvement in the SF-36 physical component exceeded the reported 7–16 points and changes in the SF-36 mental component was also at the higher end of this range^35^ .

### Effect and development over time of functional outcome in PSB standalone approach (PSB) and integrative approach (PSB+)

For strength (all angles) statistics revealed no significant time*group interaction (*p* = 0.68, η^2^p = 0.06) (Table 4) or group effects (*p* = 0.91, η^2^p = 0.0003). Postintervention, both groups showed a significant increase in strength in a linear-like manner (PSB: mean difference [95% CI] 96.79 [57.64–135.93] Nm, *p* < 0.001; PSB+ : mean difference [95% CI] 86.88 [48.70- 125.06] Nm, *p* < 0.001) (Fig. 1).

**Table 4:** Adjusted functional outcomes for the PSB and the PSB+ group.

Variables	Measurement period	PSB-only n = 20	PSB + GT + IMT n = 21	Main effect of group	Interaction effect between time and group
Strength 39° (Nm)	Baseline	173.71	173.71	*p*-value = 0.58 F = 0.31 df = 1 η^2^p = 0.008	*p*-value = 0.68 F = 0.57 df = 4 η^2^p = 0.06
	3 weeks (std. err)	197.89 (7.53)	203.06 (7.34)		
	6 weeks	229.20 (9.66)	219.43 (9.42)		
	9 weeks	247.46 (10.56)	242.23 (10.30)		
	16 weeks	271.83 (13.44)	254.40 (13.11)		
	MD (95% CI)	98.12 (58.07 to 138.17)	80.69 (41.63 to 119.76)		
	Main effect of time	*p*-value < 0.001	*p*-value < 0.001		
		F = 13.91	F = 10.92		
		df = 4	df = 4		
		η^2^p = 0.61	η^2^p = 0.56		
Strength 30° (Nm)	Baseline	175.24	175.24	*p*-value = 0.80 F = 0.07 df = 1 η^2^p = 0.002	*p*-value = 0.30 F = 1.26 df = 4 η^2^p = 0.13
	3 weeks (std. err)	195.86 (9.36)	210.56 (9.13)		
	6 weeks	231.59 (11.67)	229.78 (11.39)		
	9 weeks	248.178 (12.40)	261.98 (12.09)		
	16 weeks	270.47 (15.06)	258.36 (14.70)		
	MD (95% CI)	95.22 (50.37 to 140.11)	83.12 (39.34 to 126.91)		
	Main effect of time	*p*-value < 0.001	*p*-value < 0.001		
		F = 11.46	F = 12.65		
		df = 4	df = 4		
		η^2^p = 0.58	η^2^p = 0.59		
Strength 24° (Nm)	Baseline	181.93	181.93	*p*-value = 0.24 F = 1.41 df = 1 η^2^p = 0.04	*p*-value = 0.47 F = 0.90 df = 4 η^2^p = 0.09
	3 weeks (std. err)	196.31 (11.54)	226.08 (11.26)		
	6 weeks	225.78 (12.44)	247.50 (12.13)		
	9 weeks	250.94 (11.73)	264.97 (11.45)		
	16 weeks	267.52 (14.56)	271.50 (14.21)		
	MD (95% CI)	85.59 (42.19 to 129.00)	89.58 (47.23 to 131.93)		
	Main effect of time	*p*-value < 0.001	*p*-value < 0.001		
		F = 10.48	F = 12.71		
		df = 4	df = 4		
		η^2^p = 0.55	η^2^p = 0.60		
Strength 15° (Nm)	Baseline	168.54	168.54	*p*-value = 0.87 F = 0.03 df = 1 η^2^p = 0.001	*p*-value = 0.99 F = 0.08 df = 4 η^2^p = 0.01
	3 weeks (std. err)	205.56 (12.51)	211.13 (12.20)		
	6 weeks	229.74 (14.21)	235.48 (13.85)		
	9 weeks	254.28 (14.09)	257.45 (13.74)		
	16 weeks	271.27 (15.49)	267.86 (15.10)		
	MD (95% CI)	102.73 (56.58 to 148.89)	99.35 (54.34 to 144.35)		
	Main effect of time	*p*-value < 0.001	*p*-value < 0.001		
		F = 11.25	F = 11.44		
		df = 4	df = 4		
		η^2^p = 0.56	η^2^p = 0.58		
Strength (all angles) (Nm)	Baseline	174.85	174.85	*p*-value = 0.91 F = 0.01 df = 1 η^2^p = 0.0003	*p*-value = 0.68 F = 0.58 df = 4 η^2^p = 0.06
	3 weeks (std. err)	200.37 (8.74)	211.32 (8.52)		
	6 weeks	230.60 (10.53)	231.59 (10.27)		
	9 weeks	251.70 (10.92)	255.23 (10.65)		
	16 weeks	271.64 (13.13)	261.74 (12.81)		
	MD (95% CI)	96.79 (57.64 to 135.93)	86.88 (48.70 to 125.06)		
	Main effect of time	*p*-value < 0.001	*p*-value < 0.001		
		F = 14.33	F = 14.04		
		df = 4	df = 4		
		η^2^p = 0.62	η^2^p = 0.62		

### Correlation analysis between changes in the primary and secondary outcomes

A statistically significant correlation was found between multifidus CSA change and ODI change in the PSB+ group (r = 0.57, *p* = 0.001) but not in the PSB group (r = 0.01, *p* = 0.961). Moderate correlations were found for CSA change and VAS change (r = 0.48, *p* = 0.007) and SF physical (r = 0.4, *p* = 0.034) in the PSB+ group, but not in the PSB-group (VAS: r = − 0.25, *p* = 0.20; SF-P: r = 0.22, *p* = 0.25). There were no statistically significant Pearson correlations between the secondary outcomes and MT (Table 5).

**Table 5:** Correlations between changes in multifidus CSA and MT and changes in secondary outcomes.

Group	Δ VAS	Δ ODI	Δ SF-P	Δ SF-M
PSB				
Δ CSA	− 0.25	0.01	0.22	0.06
Δ MT	0.03	0.06	0.05	0.06
Δ Strength	0.05	0.30	0.39	− 0.02
PSB+				
Δ CSA	0.48**	0.57**	0.4**	0.23
Δ MT	− 0.20	0.21	0.15	− 0.18
Δ Strength	− 0.20	0.19	0.14	− 0.17

**indicates *p* < 0.01.

## Discussion

### Summary of the results

This is the first study to closely monitor the individual morphological, functional, and clinical effects of ILEX throughout the course of a 16-week intervention, while demonstrating its safety in a cohort with clear indications of nociceptive and/or neuropathic pain (e.g., disk herniations and radiculopathies). The study aimed to (1) closely monitor and analyze the effects of ILEX applied as a stand-alone intervention and (2) to compare this approach with an integrative ILEX program that includes GE and MT. Both the stand-alone approach and the integrative approach showed meaningful improvements across structural, functional and patient-reported outcomes from the very beginning of the 16-week intervention period, with largely comparable responses between the groups. Multifidus CSA increased substantially in both groups, indicating a robust hypertrophic response to the core ILEX program, while MT showed small gains and EI remained largely unchanged. Pain (VAS) and disability (ODI) improved in both groups, with reductions exceeding MCID, and physical and mental quality of life also increased over time. Isometric extension strength improved markedly in both groups, with negligible differences between them. Overall, the results suggest that the core ILEX program accounted for the majority of observed improvements, and the addition of GE and MT did not confer a clear or reliably detectable benefit.

### Contextualization

Regarding our primary outcome, multifidus CSA, Fortin et al. investigated ILEX combined with motor control strategies versus general exercise (without ILEX) in nonspecific LBP and found that the ILEX and motor control combination was more effective in restoring muscle size^20^. Our study, which did not include additional motor control techniques, demonstrates that similar CSA gains can be achieved with ILEX alone. Notably, motor control was embedded in our protocol through software-based biofeedback, guiding patients to modulate muscle activation across a controlled flexion–extension cycle (10s total: 4s in each direction plus a 2s isometric hold). Regarding secondary outcomes, our findings support previous evidence that ILEX induces significant functional improvements. As Steele et al. argued in their review, improvements in lumbar extension strength are correlated with reductions in low back pain^16^. Although both of our groups demonstrated a linear progressive increase in strength, correlation analysis did not reveal a significant relationship, leaving room for the possibility that additional factors may contribute to clinical improvements. Only few studies have investigated the effects of ILEX in cohorts beyond nonspecific LBP, such as patients presenting with radicular symptoms or nerve root compressions, similar to our patient population. In a previous retrospective study from our group, Golonka et al. reported that 162 of 168 patients (96,4%) who underwent the same multimodal program- including general exercise (GE) and manual therapy (MT)- experienced significant symptomatic improvements^19^. Our prospective intervention demonstrates that comparable clinical outcomes can be achieved with ILEX alone. Supporting this, a recent study by Kumar et al. (n = 15 per group) found that ILEX was more clinically effective than traditional core stabilization exercises (e.g., elbow bridging, hip rotations, crunches) in patients with radiologically confirmed lumbar disc herniations^36^. In sum, there is considerable evidence that specifically targeting the multifidus represents a key mechanism in the resolution of LBP.

### Mechanisms

In the search for possible explanations and underlying mechanisms, this study contributes to the current literature by integrating functional, structural, and clinical outcomes. While muscle deconditioning in the form of atrophy and fat infiltration has long been hypothesized as a key mechanism in nonspecific low back pain^8^, recent research has increasingly emphasized factors relevant to optimal paraspinal muscle function, such as multifidus dysfunction^6,7^. In cohorts where neural pathways are involved and nerve compression (e.g., through lumbar disc herniations) may contribute not only to structural muscle deconditioning but also to functional impairments via arthrogenic and reflex inhibition, interventions that actively engage the muscles may be particularly effective. Although attempts have been made to electrically stimulate the multifidus via implants^7^, our closely monitored design revealed that a systematic ILEX protocol starting slowly with low resistance and incorporating biofeedback-controlled motor control- may be crucial to first restore muscle function, before progressively increasing resistance to promote hypertrophy. Thus, long-term resolution of LBP in this cohort may depend initially on reactivating muscle function, with structural restoration following subsequently. However, it remains unclear whether muscle size and quality can be fully restored. In our study, echo intensity (EI) levels did not decrease, consistent with Fortin et al., who also observed no change in muscle quality^20^. To date, only Welch et al. reported a definitive reduction in fat infiltration^37^, suggesting that structural qualitative improvements may be more difficult to achieve than functional gains. One suggestion would therefore be to implement even longer intervention periods or to further increase training frequency and volume^20^. On a critical note, several aspects relevant for long-term rehabilitation were not addressed in our study, including the potential for fiber-type transformation, changes in fascial stiffness, modulation of inflammatory processes, and other factors that may influence recovery, as these cannot be assessed with ultrasound.

### Strengths

A major strength of this study is its comprehensive design, which simultaneously assessed morphological, functional, and clinical outcomes in a real-world clinical setting. The closely monitored assessment schedule allowed detection of early adaptations during the low-intensity phase as well as gains during the subsequent higher-intensity strengthening phase, demonstrating a linear-like progression of improvements. Unlike most studies, focusing on nonspecific LBP^15,16^, our participants presented clinically relevant diagnoses and symptoms, often having undergone multiple prior interventions, including physiotherapy, massage, or even with relative indications for surgery. Many patients were fragile, with advanced deconditioning, and were unable to perform other recommended exercises, such as yoga or conventional core stabilization. Despite this, the intervention was highly effective, with only a single patient ultimately requiring surgery. Furthermore, the intervention was delivered using modern ILEX equipment, which allowed precise adjustment of both resistance and range of motion tailored to each patient’s current condition, diagnoses, and pain perception. This systematic approach minimized flare-ups while allowing progressive overload and contributed to remission in patients with structural deficits. Adverse events manifesting as sustained increases in pain were promptly managed by temporarily slowing the progression of overload or, if necessary, reducing resistance until pain subsided. These factors highlight the safety and suitability of the protocol even for fragile and previously untrainable patients and is a crucial step towards therapy individualization. The feasibility and acceptability of this protocol is further underlined with the high patient attendance rates. This systematically structured approach facilitated adherence and engagement, even among patients with complex, chronic or otherwise refractory patients.

### Limitations

Our results are specific to our patient cohort and may not be generalizable to other conditions or populations. For instance, robust evidence supports the effectiveness of MT and related soft tissue techniques in chronic neck pain^38^. In our study, if our hypothesis is confirmed -that ILEX and targeted muscle conditioning are highly relevant for addressing the key drivers of nociceptive and neuropathic pain- it remains unclear how these findings translate to other LBP cohorts characterized predominantly by nociplastic pain^18^. In such cases, components of MT relevant to education and pain awareness may play a more central role by fostering long-term behavioral and cognitive changes that influence long-term therapeutic success^26,39^ In addition, the total number of MT sessions (mean 6.84 ± 1.77) was considerably lower than the number of ILEX sessions (mean 23.13 ± 0.81). It is therefore unclear whether a higher dose of MT might have further improved outcomes, particularly in patients requiring greater biopsychosocial support^4,39,40^. Similarly, the GE were performed as a single set only. Performing multiple sets for these bigger muscle groups might enhance their effectiveness, and additional GE or MT could have a positive impact on functional outcomes not captured by the ILEX-specific strength test.

Furthermore, randomization was not feasible due to organizational constraints; patients self-selected between the multimodal ILEX approach and ILEX stand-alone, introducing the possibility of selection bias. The dropout rate of 13.7% (n = 10) slightly exceeded typical rates at the facility (roughly 10%), with a higher dropout in the ILEX-only group (n = 6) compared to the multimodal ILEX-group (n = 4), suggesting that compliance may differ depending on treatment format. Our total sample size (n = 58) was comparable to Fortin et al. (n = 50)^20^, and post-hoc calculations indicated that a smaller number (n = 44, excluding anticipated dropouts) would have been sufficient to achieve adequate statistical power. Regarding between-group comparisons and the effect of time, the small effect sizes in the main outcomes do not indicate a clear advantage of the integrative program over the stand-alone ILEX approach. Both groups, however, showed marked improvements over time in clinical, functional and most morphological measures. For some outcomes with wide confidence intervals, particularly muscle thickness (MT) and echo intensity (EI), as well as clinical outcomes such as the Oswestry Disability Index (ODI), the evidence remains less precise, leaving some statistical uncertainty. Other potential confounding factors include the relatively wide age range (18 to 65 years) and varying symptom characteristics due to analgesic medication or nerve irritation (numbness, tingling).

Ultrasound imaging, although increasingly used in clinical and research settings, remains inferior in resolution compared to MRI^41^. Despite experienced raters and good reliability^25,28^, image quantification is challenging, especially in degenerative muscles. The use of echo intensity (EI) as a marker of muscle quality is still debated, and its true relevance remains uncertain^42^. While higher EI has been linked to fat infiltration^43^, our study observed the highest EI in healthy controls, and EI did not change in any group, aligning with Fortin et al.^20^. This suggests that clinical improvements may occur independently of measurable changes in muscle quality. We also focused imaging on a single lumbar segment (L4/L5), corresponding to the pivot point of the ILEX device. While changes were expected primarily at this level, multifidus adaptations can occur at multiple segments depending on the underlying mechanism^41^. Consequently, potential changes at other levels may have been missed, and future studies should include multi-level imaging to capture the full extent of muscular adaptations.

While conducting the study in an authentic clinical setting provides many advantages, it also introduces several real-world limitations that may affect the results. First, patients voluntarily sought ILEX treatment at the facility on their own initiative, which could introduce selection bias, as higher expectations may influence perceived outcomes^44,45^. Despite high compliance and attendance, the longer duration of the integrative program (18.7 weeks compared to 17.0 weeks) may have been partly influenced by scheduling constraints, as sessions including GE and MT required longer time slots causing delays that may have affected outcomes. Additional factors such as holidays, illness, or other unavoidable interruptions may have further contributed to distortions. Furthermore, as in any physiotherapeutic setting, therapist-related variability may have influenced outcomes, as staff rotated collevtively during the study period. Finally, altghough patients were asked to refrain from any other therapies or programs (e.g., massages, back schools), it was not possible to fully verify adherence, leaving open the possibility that additional interventions may have occured.

### Clinical implications

Consider patient stratification and mechanism-oriented approach: The underlying pathomechanism should be carefully analyzed (e.g., in medical assessments) and taken into account when planning interventions. For example, patients with nerve root compression may particularly benefit from targeted activation and reconditioning of the paraspinal musculature, whereas a more holistic, biopsychosocial approach may be preferable when nociplastic pain predominates^18,39^. Equally important, timely adaptations across the acute, subacute and chronic stages need to be considered to ensure optimal alignment of the intervention’s goals to the patient’s condition (e.g., early muscle activation vs. long-term hypertrophy)^14^.

Implement controlled movements, systematic resistance progression and individualized range of motion (ROM): Gradually increasing resistance while ensuring proper motor control is key to restoring functional capacity and rebuilding patients’ confidence in their own ability. ROM should be tailored according to the underlying mechanism (e.g., limiting flexion in cases of nerve root compression). Therefore, ILEX interventions should ideally be delivered one-on-one by qualified personnel.

Protocol duration and progression: Structural, functional and clinical outcomes should be ideally evaluated collectively, especially at the end of the intervention. Our study suggests that 16 weeks may not fully exploit the potential of the intervention, and longer periods could yield even greater improvements.

Sustaining muscle function long-term: While follow-up data are still being analyzed and definitive conclusions cannot yet be made, regular physical activity and resistance exercise appear essential for maintaining long-term spinal health and preventing low back pain.

## Conclusion

This study demonstrates that isolated lumbar extension resistance exercise is safe and effective in improving structural functional and clinical outcomes in patients with nociceptive and/or neuropathic low back pain. Increases in cross-sectional area, muscle thickness, and lumbar extensor strength were accompanied by improvements in patient-reported outcomes, with comparable efficacy between ILEX as a stand-alone approach and as part of a multimodal program including general exercise and manual therapy. Clinical outcomes (VAS, ODI, SF-36) appear early and are maintained throughout the intervention, emphasizing the importance of targeted muscle activation and controlled progression, individualized range of motion, and adherence to protocol. Mechanism-oriented patient stratification, graded activation and long-term maintenance of muscle function appear to be key considerations for optimizing therapy. While additional modalities may be warranted in patients with predominantly nociplastic pain or complex biopsychosocial needs, the results support the use of ILEX for reconditioning the lumbar extensors and improving patient-reported outcomes.

## Supplementary Information

Below is the link to the electronic supplementary material.

Supplementary Material 1

## Data Availability

The datasets used and/or analyzed during the current study are available from the corresponding author on reasonable request.

## Abbreviations

CLBP: Chronic low back pain
CSA: Cross-sectional area
CT: Computed tomography
EI: Echo intensity
GE: General exercise
GMKT: German medical strengthening association
IFAMT: Institute for applied manual therapy
ILEX: Isolated lumbar extension resistance exercise
LBP: Low back pain
MDC: Minimal detectable change
MF: Multifidus
MRI: Magnetic resonance imaging
MT: Manual therapy
MT: Muscle thickness
ODI: Oswestry disability index
SF-36: Short form 36 survey
VAS: Visual analog scale

## References

[1] GBD 2021 Low Back Pain Collaborators. Global, regional, and national burden of low back pain, 1990–2020, its attributable risk factors, and projections to 2050: A systematic analysis of the Global Burden of Disease Study 2021. Lancet Rheumatol. 2023; **5**(6):e316–e329. 10.1016/S2665-9913(23)00098-X 10.1016/S2665-9913(23)00098-X PMC10234592 37273833

[2] Knezevic, N. N., Candido, K. D., Vlaeyen, J. W. S., Van Zundert, J. & Cohen, S. P. Low back pain. *Lancet* **398**(10294), 78–92. 10.1016/S0140-6736(21)00733-9 (2021). 34115979 10.1016/S0140-6736(21)00733-9

[3] Hartvigsen, J. et al. What low back pain is and why we need to pay attention. *Lancet* **391**(10137), 2356–2367. 10.1016/S0140-6736(18)30480-X (2018). 29573870 10.1016/S0140-6736(18)30480-X

[4] Tagliaferri, S. D. et al. Domains of chronic low back pain and assessing treatment effectiveness: A clinical perspective. *Pain Pract.* **20**(2), 211–225. 10.1111/papr.12846 (2020). 31610090 10.1111/papr.12846

[5] World Health Organization. WHO guideline for non-surgical management of chronic primary low back pain in adults in primary and community care settings. Geneva: World Health Organization. Available from: https://www.who.int/publications/i/item/9789240081789 (2023). 38198579

[6] Matheve, T., Hodges, P. & Danneels, L. The role of back muscle dysfunctions in chronic low back pain: State-of-the-art and clinical implications. *J. Clin. Med.* **12**(17), 5510. 10.3390/jcm12175510 (2023). 37685576 10.3390/jcm12175510 PMC10487902

[7] Tieppo Francio, V., Westerhaus, B. D., Carayannopoulos, A. G. & Sayed, D. Multifidus dysfunction and restorative neurostimulation: A scoping review. *Pain Med.* **24**(12), 1341–1357. 10.1093/pm/pnad098 (2023). 37439698 10.1093/pm/pnad098 PMC10690869

[8] Steele, J., Bruce-Low, S. & Smith, D. A reappraisal of the deconditioning hypothesis in low back pain: Review of evidence from a triumvirate of research methods on specific lumbar extensor deconditioning. *Curr. Med. Res. Opin.* **30**(5), 865–911. 10.1185/03007995.2013.875465 (2014). 24328452 10.1185/03007995.2013.875465

[9] Ranger, T. A. et al. Are the size and composition of the paraspinal muscles associated with low back pain? A systematic review. *Spine J.* **17**(11), 1729–1748. 10.1016/j.spinee.2017.07.002 (2017). 28756299 10.1016/j.spinee.2017.07.002

[10] Kalichman, L., Klindukhov, A., Li, L. & Linov, L. Indices of paraspinal muscles degeneration: Reliability and association with facet joint osteoarthritis: Feasibility study. *Clin. Spine Surg.* **29**(9), 465–470. 10.1097/BSD.0b013e31828be943 (2016). 27137159 10.1097/BSD.0b013e31828be943

[11] Wang, G. et al. Quantitative MRI and X-ray analysis of disc degeneration and paraspinal muscle changes in degenerative spondylolisthesis. *J. Back Musculoskelet. Rehabil.* **28**(2), 277–285. 10.3233/BMR-140515 (2015). 25096310 10.3233/BMR-140515

[12] Guven AE, Schönnagel L, Chiapparelli E, Camino-Willhuber G, Zhu J, Caffard T, et al. Relationship between Lumbar Foraminal Stenosis and Multifidus Muscle Atrophy - A Retrospective Cross-Sectional Study. Spine. 2024. [Epub ahead of print]. 10.1097/BRS.0000000000005113 10.1097/BRS.0000000000005113 39087423

[13] Fortin, M., Lazáry, Á., Varga, P. P., McCall, I. & Battié, M. C. Paraspinal muscle asymmetry and fat infiltration in patients with symptomatic disc herniation. *Eur. Spine J.* **25**(5), 1452–1459. 10.1007/s00586-016-4503-7 (2016). 26957101 10.1007/s00586-016-4503-7

[14] Hodges, P. W. & Danneels, L. Changes in structure and function of the back muscles in low back pain: Different time points, observations, and mechanisms. *J. Orthop. Sports Phys. Ther.* **49**(6), 464–476. 10.2519/jospt.2019.8827 (2019). 31151377 10.2519/jospt.2019.8827

[15] Trybulski, R. et al. Impact of isolated lumbar extension strength training on reducing nonspecific low back pain, disability, and improving function: A systematic review and meta-analysis. *Sci. Rep.* **15**, 6426. 10.1038/s41598-025-90699-5 (2025). 39984628 10.1038/s41598-025-90699-5 PMC11845604

[16] Steele, J., Bruce-Low, S. & Smith, D. A review of the clinical value of isolated lumbar extension resistance training for chronic low back pain. *PM R.* **7**(2), 169–187. 10.1016/j.pmrj.2014.10.009 (2015). 25452128 10.1016/j.pmrj.2014.10.009

[17] Gesellschaft für Medizinische Kräftigungstherapie (GMKT-D). Leitlinien (Version 2.5, 2021) [Internet]. 2021 [cited 2025 Apr 18]. Available from: https://www.gmkt.info/leitlinien

[18] Nijs, J. et al. Nociceptive, neuropathic, or nociplastic low back pain? The low back pain phenotyping (BACPAP) consortium’s international and multidisciplinary consensus recommendations. *Lancet Rheumatol.* **6**(3), e178–e188. 10.1016/S2665-9913(23)00324-7 (2024). 38310923 10.1016/S2665-9913(23)00324-7

[19] Golonka, W. et al. Isolated lumbar extension resistance exercise in limited range of motion for patients with lumbar radiculopathy and disk herniation-clinical outcome and influencing factors. *J. Clin. Med.* **10**(11), 2430. 10.3390/jcm10112430 (2021). 34070780 10.3390/jcm10112430 PMC8198576

[20] Fortin, M. et al. The Effects of combined motor control and isolated extensor strengthening versus general exercise on paraspinal muscle morphology, composition, and function in patients with chronic low back pain: A randomized controlled trial. *J. Clin. Med.* **12**(18), 5920. 10.3390/jcm12185920 (2023). 37762861 10.3390/jcm12185920 PMC10532355

[21] Owen, P. J. et al. Which specific modes of exercise training are most effective for treating low back pain? Network meta-analysis. *Br. J. Sports Med.* **54**(21), 1279–1287. 10.1136/bjsports-2019-100886 (2020). 31666220 10.1136/bjsports-2019-100886 PMC7588406

[22] Steele, J., Bruce-Low, S. & Smith, D. A review of the specificity of exercises designed for conditioning the lumbar extensors. *Br. J. Sports Med.* **49**(5), 291–297. 10.1136/bjsports-2013-092197 (2015). 24092889 10.1136/bjsports-2013-092197

[23] Faul, F., Erdfelder, E., Lang, A.-G. & Buchner, A. *G*Power 3: A flexible statistical power analysis program for the social, behavioral, and biomedical sciences. *Behav. Res. Methods* **39**, 175–191 (2007). 17695343 10.3758/bf03193146

[24] Khorami, A. K. et al. Recommendations for diagnosis and treatment of lumbosacral radicular pain: A systematic review of clinical practice guidelines. *J. Clin. Med.* **10**(11), 2482. 10.3390/jcm10112482 (2021). 34205193 10.3390/jcm10112482 PMC8200038

[25] Domokos, B. et al. Ultrasound imaging of lumbar multifidus morphology and quality—A prospective study on monitoring acute adaptations to specific exercise and on reliability. *J. Orthop. Sports Phys. Ther. Open* **2**(4), 354–363 (2024).

[26] Institut für Angewandte Manuelle Therapie (IFAMT) [Internet]. Applied Manual Therapy. [cited August 2025]. Available from www.ifamt.de.

[27] Graves, J. E. et al. Pelvic stabilization during resistance training: its effect on the development of lumbar extension strength. *Arch. Phys. Med. Rehabil.* **75**(2), 210–215 (1994). 8311680

[28] Koppenhaver, S. L., Hebert, J. J., Parent, E. C. & Fritz, J. M. Rehabilitative ultrasound imaging is a valid measure of trunk muscle size and activation during most isometric sub-maximal contractions: A systematic review. *Aust. J. Physiother.* **55**(3), 153–169. 10.1016/s0004-9514(09)70076-5 (2009). 19681737 10.1016/s0004-9514(09)70076-5

[29] Price, D. D., McGrath, P. A., Rafii, A. & Buckingham, B. The validation of visual analogue scales as ratio scale measures for chronic and experimental pain. *Pain* **17**(1), 45–56. 10.1016/0304-3959(83)90126-4 (1983). 6226917 10.1016/0304-3959(83)90126-4

[30] Fairbank, J. C. & Pynsent, P. B. The oswestry disability index. *Spine* **25**(22), 2940–2952. 10.1097/00007632-200011150-00017 (2000). 11074683 10.1097/00007632-200011150-00017

[31] Ware, J. E. & Sherbourne, C. D. The MOS 36-item short-form health survey (SF-36): I. Conceptual framework and item selection. *Med. Care* **30**(6), 473–483. 10.1097/00005650-199206000-00002 (1992). 1593914

[32] Robinson, M. E., Greene, A. F., O’Connor, P., Graves, J. E. & Mac, M. M. Reliability of lumbar isometric torque in patients with chronic low back pain. *Phys. Ther.* **72**(3), 186–190. 10.1093/ptj/72.3.186 (1992). 1533939 10.1093/ptj/72.3.186

[33] Machin, D., Campbell, M. J., Tan, S. B. & Tan, S. H. *Sample Size Tables for Clinical Studies* (John Wiley & Sons, 2009).

[34] Olsen, M. F. et al. Minimum clinically important differences in chronic pain vary considerably by baseline pain and methodological factors: Systematic review of empirical studies. *J. Clin. Epidemiol.* **101**, 87-106.e2. 10.1016/j.jclinepi.2018.05.007 (2018). 29793007 10.1016/j.jclinepi.2018.05.007

[35] Lauridsen, H. H., Hartvigsen, J., Manniche, C., Korsholm, L. & Grunnet-Nilsson, N. Responsiveness and minimal clinically important difference for pain and disability instruments in low back pain patients. *BMC Musculoskelet. Disord.* **7**, 82. 10.1186/1471-2474-7-82 (2006). 17064410 10.1186/1471-2474-7-82 PMC1635558

[36] Kumar, P. et al. Effectiveness of isolated lumbar extension resistance and core stabilization exercise on lumbar disc herniation. *Int. J. Hum. Mov. Sports Sci.* **13**(3), 583–589. 10.13189/saj.2025.130312 (2025).

[37] Welch, N. et al. The effects of a free-weight-based resistance training intervention on pain, squat biomechanics and MRI-defined lumbar fat infiltration and functional cross-sectional area in those with chronic low back pain. *BMJ Open Sport Exerc. Med.* **1**(1), e000050. 10.1136/bmjsem-2015-000050 (2015). 27900136 10.1136/bmjsem-2015-000050 PMC5117021

[38] Wang, S. Q., Jiang, A. Y. & Gao, Q. Effect of manual soft tissue therapy on the pain in patients with chronic neck pain: A systematic review and meta-analysis. *Complement. Ther Clin Pract.* **49**, 101619. 10.1016/j.ctcp.2022.101619 (2022). 35988324 10.1016/j.ctcp.2022.101619

[39] Grenier, J. P. & Rothmund, M. A critical review of the role of manual therapy in the treatment of individuals with low back pain. *J. Man. Manip. Ther.* **32**(5), 464–477. 10.1080/10669817.2024.2316393 (2024). 38381584 10.1080/10669817.2024.2316393 PMC11421166

[40] Fleckenstein, J. et al. Individualized exercise in chronic non-specific low back pain: A systematic review with meta-analysis on the effects of exercise alone or in combination with psychological interventions on pain and disability. *J. Pain* **23**(11), 1856–1873. 10.1016/j.jpain.2022.07.005 (2022). 35914641 10.1016/j.jpain.2022.07.005

[41] Hodges, P. W., Bailey, J. F., Fortin, M. & Battié, M. C. Paraspinal muscle imaging measurements for common spinal disorders: review and consensus-based recommendations from the ISSLS degenerative spinal phenotypes group. *Eur. Spine J.* **30**(12), 3428–3441. 10.1007/s00586-021-06990-2 (2021). 34542672 10.1007/s00586-021-06990-2

[42] Wong, V. et al. Exercise induced changes in echo intensity within the muscle: A brief review. *J. Ultrasound* **23**(4), 457–472. 10.1007/s40477-019-00424-y (2020). 31925731 10.1007/s40477-019-00424-y PMC7588570

[43] Yoshiko, A. et al. Muscle quality characteristics of muscles in the thigh, upper arm and lower back in elderly men and women. *Eur. J. Appl. Physiol.* **118**(7), 1385–1397. 10.1007/s00421-018-3870-7 (2018). 29687267 10.1007/s00421-018-3870-7

[44] Saueressig, T. et al. The importance of context (placebo effects) in conservative interventions for musculoskeletal pain: A systematic review and meta-analysis of randomized controlled trials. *Eur. J. Pain* **28**(5), 675–704. 10.1002/ejp.2222 (2024). 38116995 10.1002/ejp.2222

[45] Wassinger, C. A. et al. The role of patient recovery expectations in the outcomes of physical therapist intervention: A systematic review. *Phys. Ther.* **102**(4), pzac008. 10.1093/ptj/pzac008 (2022). 35224644 10.1093/ptj/pzac008

