# Supplementary material for: Isolated lumbar extension exercise alone or in a multimodal program for low back pain and radiculopathy: a non-randomized controlled trial
Source: Sci Rep. 2025 Oct 23;15:37157. doi: 10.1038/s41598-025-22452-x (PMC12550044; doi:10.1038/s41598-025-22452-x)
Supplement: Supplementary file 1 — Supplementary Material 1 [file 41598_2025_22452_MOESM1_ESM.docx]

**Isolated Lumbar Extension Exercise Alone or in a Multimodal Program for Low Back Pain and Radiculopathy: A Non-Randomized Controlled Trial**

^1,2^ Domokos, Bruno
^2^ Domokos, Julia

^3,4^ Andersson, Gustav

^5^ Mannel, Stefan

^2^ Weigel, Linda May

^6^ Koch, Horst Josef

^7^ Wallmann-Sperlich, Birgit
^1^ Raschka, Christoph
^1,2,^ Spang, Christoph

**Appendix**

**Figure A1.** Diagrammatic representation of the ILEX lumbar machine (left) and image of Powerspine Back (PSB) (right)

| *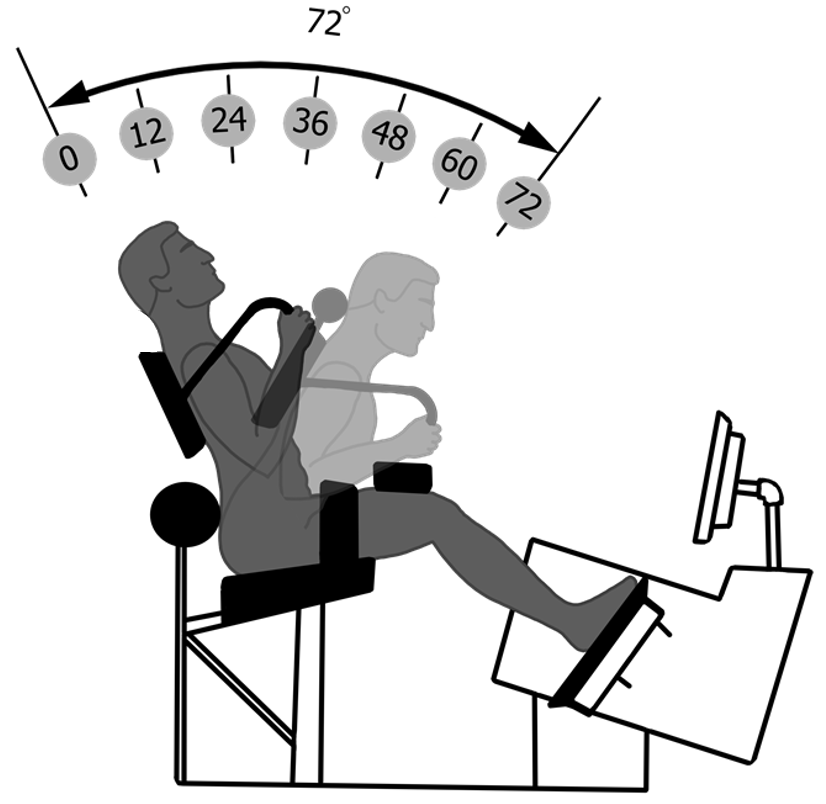* | 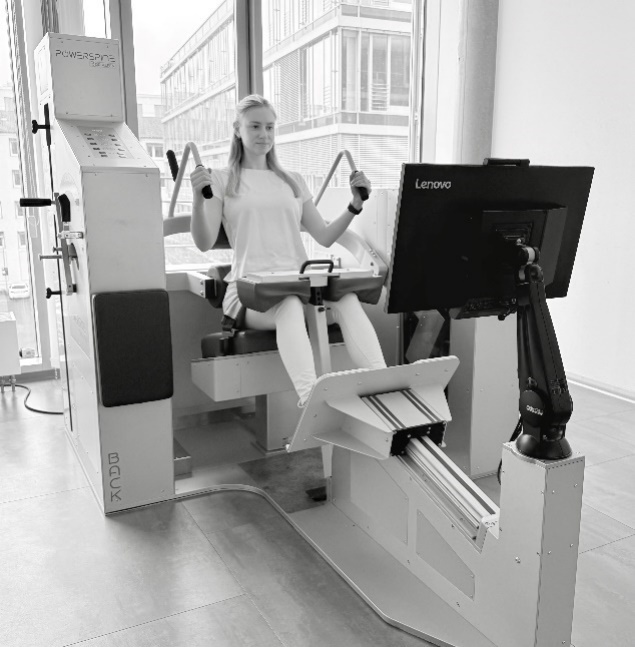 |
| --- | --- |

**Figure A2.** Consort 2025 flow diagram (Hopewell et al., 2025; modified)

**
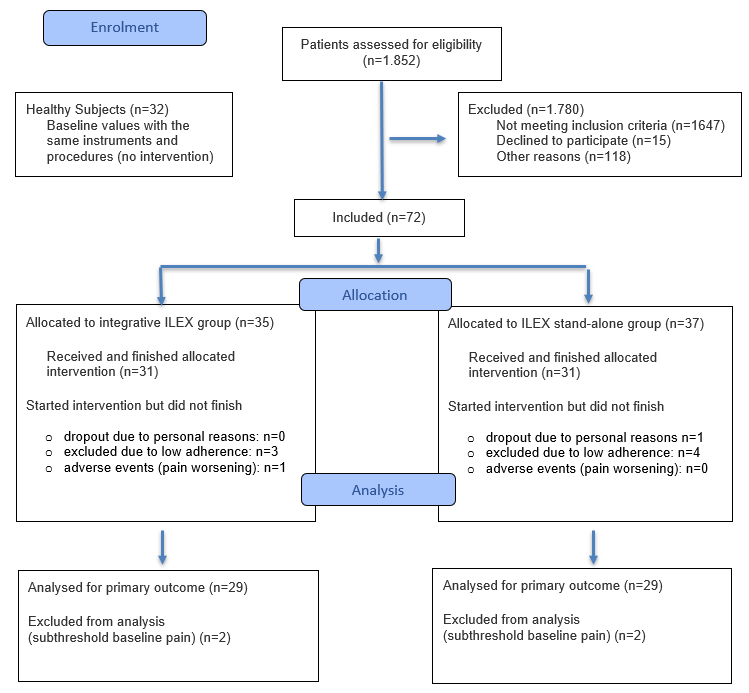
**

**Figure A3.** Histograms of absolute changes in outcome measures per group (frequency).

| **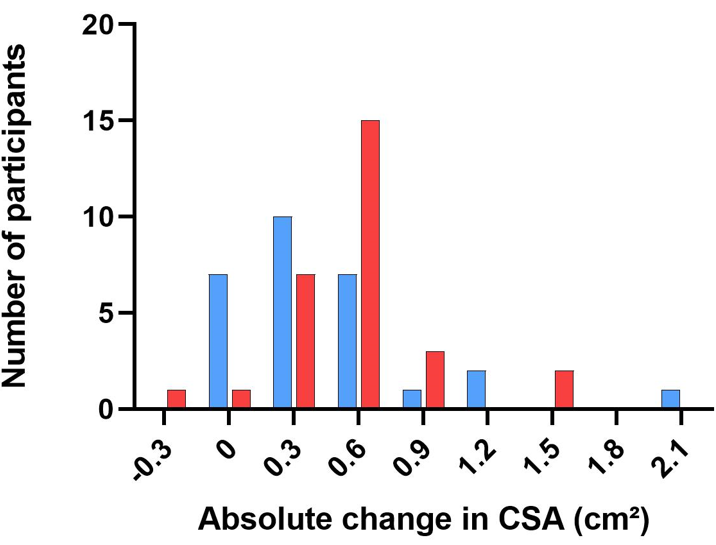** | **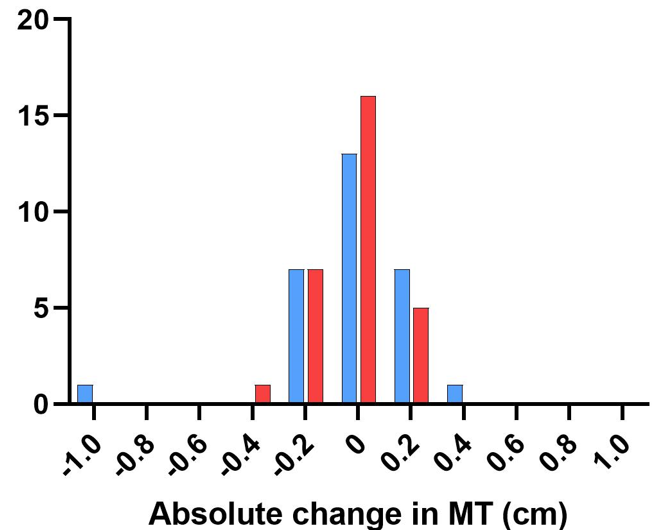** |
| --- | --- |
| **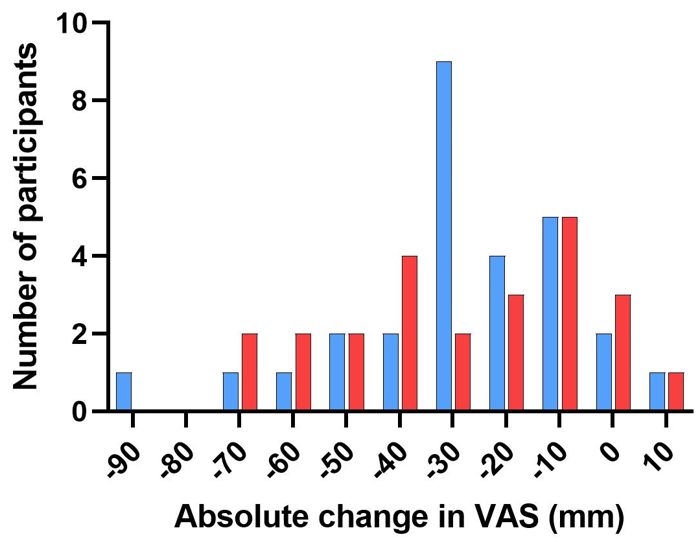** | **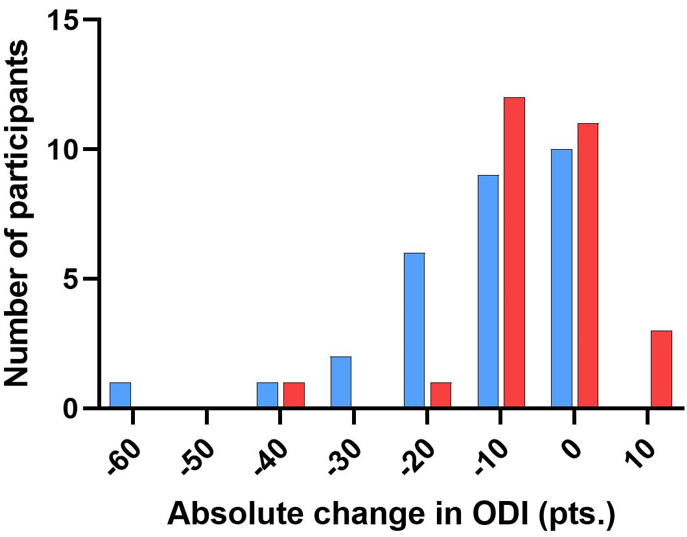** |
| **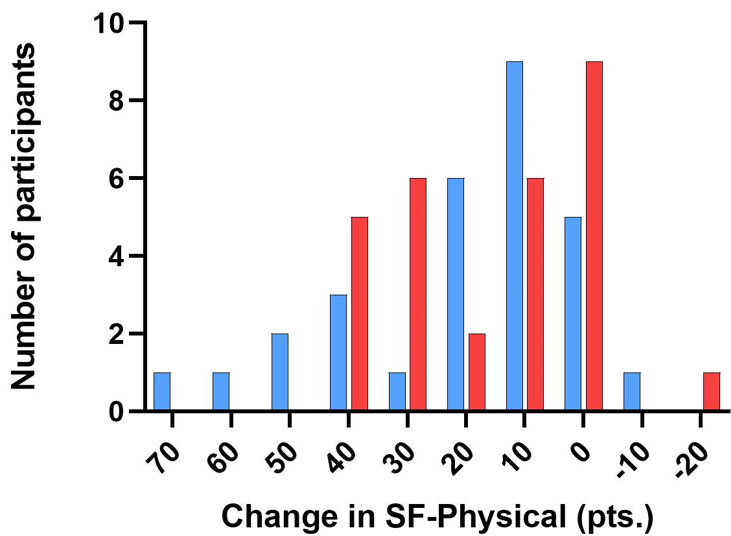** | **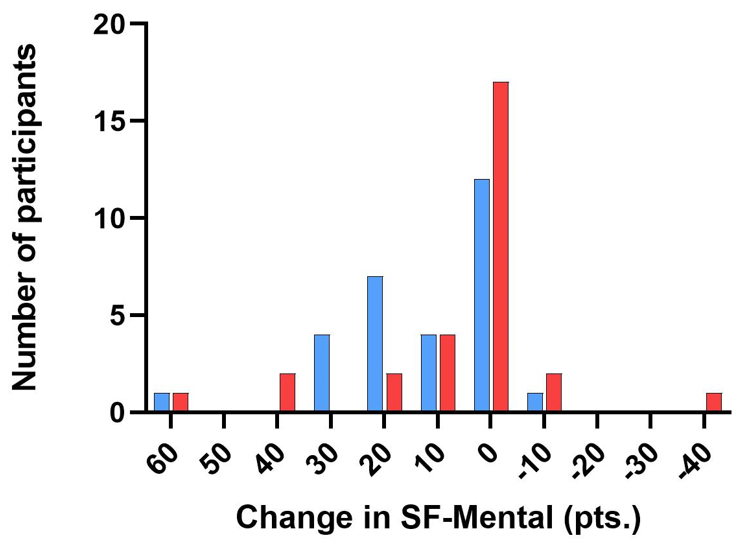** |
| **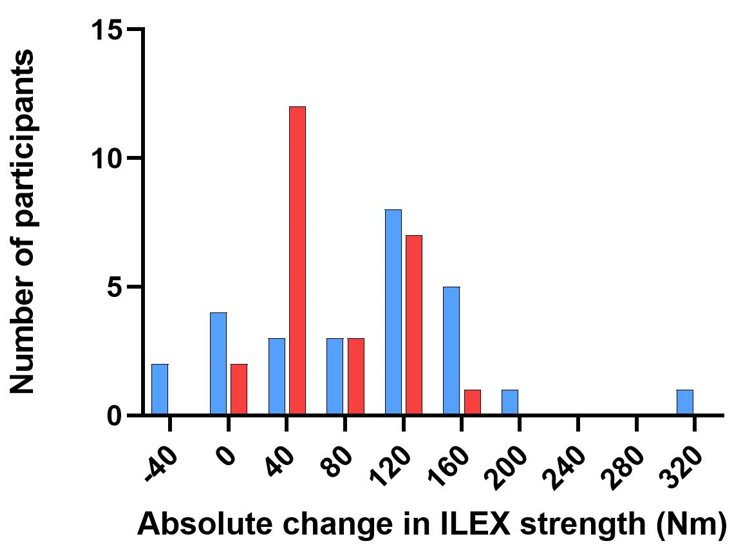** | **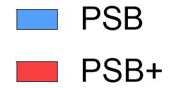** |

**Table A1.** Detailed inclusion and exclusion criteria for participants defined in the clinical assessment.

| **Inclusion Criteria** | **Exclusion Criteria** |
| --- | --- |
| - Symptoms located between gluteal fold and lower thoracic spine, including leg pain associated with nerve root compression due to lumbar disc herniation or other spinal pathologies (e.g., spinal spondylolisthesis, spondylarthritis) - Pain duration > 3 months, with no other primary pain (e.g., neck pain) - Mild to moderate pain intensity (VAS 25-75 at clinical assessment) - Age between 18 and 65 years | **Red-Flag Conditions (spine-specific):**   - Spinal tumour / metastases - Acute vertebral fracture or spinal instability - Spinal infections (e.g., spondylodiscitis, abscess) - Cauda-equina syndrome - Large aortic aneurysm affecting lumbar spine - Severe progressive neurological deficits   **Other exclusion criteria:**   - Acute back injury (< 6 weeks) - Severe cardiovascular disease (< 75 watts during standardized bicycle ergometry) - Osteoporosis with high fracture risk - Active rheumatological disease - Pregnancy - Recent spinal surgery (< 6-12 months) - Neurological disorders affecting motor control (e.g. multiple sclerosis, stroke) - Active infection - Cognitive or language limitations preventing safe training |

**Table A2.** List of general exercises in the PSB+ group. Depending on the condition, the patient performed four of these exercises during one session.

| **Exercise** | **Explanation** |
| --- | --- |
| Bilateral Proprioceptive Cable Pull | The patient stands on a soft surface with the ankle, knee and hip joints slightly bent. The upper body is slightly leaned forward, and the back is kept in a neutral position. The patient pulls the cables in front with extended arms, drawing them towards the hips while performing scapular retraction. The goal of the exercise is to train postural control and strengthen the core muscles and the upper back (latissimus dorsi, rhomboids, rear deltoids). |
| Bilateral Cable Core Stabilization | The patient lies on the back with the knees bent and feet on the ground while the toes are pointing upwards. The pelvis is tilted posteriorly to activate the core muscles. The patient pulls the cables from overhead with extended arms, bringing them down to the thighs, while resisting the lever effect of the cable. |
| Bilateral Lat Pulldown | The patient performs a bilateral latissimus pulldown while seated and secured on a chair. The upper body is slightly leaned forward, and the arms are pulled down evenly and in a controlled manner. The goal is to strengthen the upper back muscles (latissimus dorsi, trapezius, rhomboids) and to extend and decompress the spine. |
| Bilateral Rowing | The patient sits on a chair and leans the upper body forward against a support pad. A rowing movement is performed, ensuring that the movement engages not only the arms but also the back muscles (latissimus dorsi, rhomboids) by pro- and retracting the scapular, while maintaining a neutral lumbar spine. In addition to posture training, this exercise primarily targets the activation and strengthening of the upper back. |
| Seated Dips | The patient sits securely strapped on a chair and performs a downward pressing movement with the arms from a slightly leaned-forward position until the arms are nearly fully extended. Special focus is placed on postural control (maintaining a neutral spine), along with simultaneous scapular depression and retraction. The goal is to promote spinal extension and decompression while improving posture and activating the core-stabilizing muscles. |
| Abdominal Crunch | The patient sits in an abdominal machine and performs a rounded forward movement of the upper body against a cushion. The aim is to achieve targeted activation and strengthening of the abdominal muscles. |

**Table A3.** Anthropometric data and baseline values of patient groups and healthy individuals

| **Variables** | **PSB** | **PSB +** | **Healthy group** | **p-value** |
| --- | --- | --- | --- | --- |
| Age (y) | 40.26 (± 13.71) | 42.00 (± 12.69) | 35.11 (± 12.83) | 0.39 |
| Height (m) | 1.73 (± 0.11) | 1.76 (± 0.09) | 1.75 (± 0.10) | 0.19 |
| Weight (kg) | 75.87 (± 16.44) | 78.48 (± 13.38) | 73.82 (± 13.00) | 0.54 |
| BMI (kg/m^2^) | 25.16 (± 4.07) | 25.20 (± 3.29) | 23.98 (± 3.02) | 0.41 |
| VAS (mm) | 49.05 (± 19.44) | 46.45 (± 25.10) | - | - |
| ODI (pts.) | 23.90 (± 14.85) | 19.90 (± 9.76) | - | - |
| SF Physical (pts.) | 55.52 (± 15.58) | 57.16 (± 22.25) | 88.50 (± 11.20) | < 0.001*** |
| SF Mental (pts.) | 60.88 (± 21.84) | 67.36 (± 18.17) | 78.47 (± 16.65) | < 0.001*** |
| CSA (cm²) | 7.93 (± 1.79) | 8.07 (± 1.45) | 7.27 (± 1.62) | 0.126 |
| MT (cm) | 3.09 (± 0.58) | 3.17 (± 0.51) | 3.06 (± 0.46) | 0.729 |
| EI (AU) | 73.34 (± 16.70) | 67.32 (± 10.03) | 77.24 (± 11.56) | 0.015* |
| Strength 39° (Nm) | 166.21 (± 67.34) | 180.89 (± 71.21) | 205.59 (± 84.20) | 0.127 |
| Strength 30° (Nm) | 171.68 (± 66.20) | 181.07 (± 60.87) | 207.75 (± 86.93) | 0.144 |
| Strength 24° (Nm) | 177.89 (± 71.77) | 183.74 (± 67.44) | 210.50 (± 86.93) | 0.197 |
| Strength 15° (Nm) | 158.11 (± 71.49) | 179.74 (± 76.82) | 215.22 (± 93.54) | 0.028* |

*Mean difference is significant at the 0.05 level, *** at the 0.001 level
